# Diseases of the Eye

**Published:** 1891-02

**Authors:** 


### Book Reviews.

DISEASES OF THE EYE. By Edward Kettleship, F. R. C. S., Ophthalmic Surgeon to St. Thomas' Hospital; Surgeon to the Royal London (Moorfields) Ophthalmic Hospital; Late Ophthalmic Surgeon to the Hospital for sick Children, Great Ormond street. Fourth American from the Fifth English Edition. With a Chapter on Examination for Color Perception. By William Thomson, M. D., Professor of Ophthalmology in the Jefferson Medical College of Philadelphia, Published by Lea Bros. & Co., Philadelphia, 1890.

This little work of Edward Nettleship, is the best arranged and most satisfactory book on the diseases of the eye, we have ever seen. It is a real pleasure to read it, on account of its clearness and simplicity. We cheerfully commend it to the profession. A. W. G.

